# Tissue resident stem cells: till death do us part

**DOI:** 10.1007/s10522-013-9469-9

**Published:** 2013-10-02

**Authors:** Hadas Raveh-Amit, Sara Berzsenyi, Virag Vas, Danna Ye, Andras Dinnyes

**Affiliations:** 1 Biotalentum Ltd, Godollo, 2100 Hungary; 2 Molecular Animal Biotechnology Laboratory, Szent Istvan University, Godollo, 2100 Hungary; 3 Department of Farm Animal Health, Faculty of Veterinary Medicine, Utrecht University, 3584 CL Utrecht, The Netherlands

**Keywords:** Adult stem cells, Aging, Regeneration, Age-related diseases, Stem cell therapy

## Abstract

Aging is accompanied by reduced regenerative capacity of all tissues and organs and dysfunction of adult stem cells. Notably, these age-related alterations contribute to distinct pathophysiological characteristics depending on the tissue of origin and function and thus require special attention in a type by type manner. In this paper, we review the current understanding of the mechanisms leading to tissue-specific adult stem cell dysfunction and reduced regenerative capacity with age. A comprehensive investigation of the hematopoietic, the neural, the mesenchymal, and the skeletal stem cells in age-related research highlights that distinct mechanisms are associated with the different types of tissue stem cells. The link between age-related stem cell dysfunction and human pathologies is discussed along with the challenges and the future perspectives in stem cell-based therapies in age-related diseases.

## Introduction

Adult stem cells, or resident stem cells, are undifferentiated cells found in adults throughout life (Passier and Mummery 2003). These cells are characterized by two developmental capacities: *self*-*renewal*, the ability to give rise to daughter stem cells, and *multipotency*, the capacity to differentiate into various cell types of a particular organ. These properties provide stem cells the ability to sustain their population during life, and to maintain tissue remodeling and repair (Bryder et al. 2006), facilitating the rationale for using adult stem cells in regenerative medicine (Mimeault et al. 2007). Nevertheless, it is still under continuous debate: which type of stem cells is most suitable for regenerative therapy; the embryonic stem cells, the induced pluripotent stem cells (iPSCs), or the adult stem cells (Abramovich et al. 2008; Passier and Mummery 2003; Robinton and Daley 2012; Symonds et al. 2009; Yamanaka 2009).

Stem cell function declines with age and this occurs at multiple levels, including self-renewal and differentiation potential, leading to reduced regenerative capacity of all tissues and organs. Moreover, age-related alterations in stem cells contribute to distinct pathophysiological characteristics depending on the tissue of origin and function. Thus, given the perspectives of adult stem cells in regenerative therapy, understanding these processes and their role in age-related diseases (ARDs) is vital.

Here, we present a comprehensive overview of recent achievements and debates about the dysregulation of four types of tissue stem cells in the elderly (hematopoietic, neural, mesenchymal, and skeletal stem cells), highlighting that distinct age-related effects are associated with the different types of tissue stem cells. We analyze how the dysfunction of the tissue stem cells and the decrease in regeneration potential are associated with age and ARDs (Table 1). Last but not least, we explore recent progress and challenges of stem cell-based therapies in light of current progress in the field of aging. The cellular hallmarks of aging and the intrinsic pathways leading to stem cell dysfunction with age are fundamental and relevant issues, but are beyond the scope of this paper and reviewed elsewhere (Moskalev et al. 2012; Lopez-Otin et al. 2013; Tacutu et al. 2011).

**Table 1:** Major age-related changes in adult stem cells and their association with age-related diseases (ARDs)

Adult stem cell source	Major changes during aging	Stem cells in ARDs	Selected refs
HSCs (hematopoietic stem cells)	Increased HSC pool in the bone marrow (BM)	Age-associated alterations in HSCs may contribute to increased leukemia incidence with age and to favor the emergence of ARDs, including non-hematopoietic cancers and UAE	(Henry et al. 2011; Rossi et al. 2008; Van Zant and Liang 2012)
	Lymphoid to myeloid lineage shift		
	Reduced homing ability to the BM upon transplantation	HSC transplantation was suggested as an alternative approach for the treatment of age-related hematological malignancies	
	Structural changes in the BM niche		
NSCs (neural stem cells)	Reduced NSC proliferation	NSC aging evidenced by reduced neurogenesis may contribute to the progression of neurodegenerative disease	(Artegiani and Calegari 2012; Riddle and Lichtenwalner 2007)
	Reduced neurogenesis		
	Altered cell signaling in the niche	NSC transplantation to replace damaged/lost tissues or to induce endogenous neurogenesis might be used in the future to slow down age-related cognitive decline	
MSCs (mesenchymal stem cells)	Reduced MSC pool	MSC aging may contribute to the progression of ARDs, for example osteoarthritis	(Stolzing et al. 2008; Alt et al. 2012)
	Altered differentiation potential		
	Altered systemic environment	MSC transplantation was suggested as an alternative approach for the treatment of ARDs, including diabetes, cardiovascular and neurodegenerative diseases	
Skeletal muscle stem cells	Reduced satellite pool?^a^	The contribution of skeletal stem cell aging to ARDs is not clear	(Shadrach and Wagers 2011; Briggs and Morgan 2013; Pannerec et al. 2012; Yablonka-Reuveni 2011)
	Myogenic to fibrogenic lineage shift	Satellite cell transplantation may provide a strategy to slow the progression of muscular dystrophies	
	Altered cell signaling in the niche		

*UAE* unexplained anemia in the elderly

^a^Subject under debate (detailed in “Pool of satellite cells” section)

## Hematopoietic stem cells

Hematopoietic stem cells (HSCs), first identified in the 1960s (Becker et al. 1963; Till and Mc 1961), are morphologically small cells, with high nucleus to cytoplasm ratio and are defined immunophenotypically by a distinct surface marker expression profile. Human HSCs do not express the markers of differentiated blood lineages (lin minus cells) but are characterized by CD34/CD90 positivity and CD38/CD45RA negativity. Recently, the identification of the integrin molecule CD45f provided a means to isolate the long-term engrafting multipotent HSC population. Based on these markers, the number and the frequency of HSCs were easily measured, and cells were purified for further analysis from different age groups (Notta et al. 2011).

### Age-related changes in HSCs

In terms of the age-related alterations, HSCs are the most understood cells when compared to other types of tissue stem cells. With age, an overall functional decline in the hematopoietic system is characterized by reduced lymphoid cell and increased myeloid cell population with attenuated immune functions. Thus, age-related changes in the blood system are at least partially attributed to HSCs that reside in the bone marrow microenvironment and are responsible for the continuous blood cell supply (Woolthuis et al. 2011).

#### HSC pool and differentiation potential

With age, the bone marrow undergoes structural changes, such as the reduced proportion of active blood-forming bone marrow and the decreased overall cellularity. At a young age, the hematopoiesis takes place in the cavities of long bones, however, during adulthood it becomes restricted to certain bones, including the sternum, the vertebrae and the pelvis (Pearce et al. 2007). According to a long-standing paradigm, the mechanism underlying this phenomenon is the reduced HSC pool in aged individuals. However, based on more accurate stem cell purification methods, it has been recently shown that in humans the HSC population rather increases with age (Kuranda et al. 2011; Pang et al. 2011). The well-studied (C57BL/6) mouse HSC system provided an elegant model to resolve the “decreased blood cell function versus increased HSC pool” discrepancy (Muller-Sieburg et al. 2012). It was concluded that the HSC population is heterogeneous and different stem cells sub-populations with distinct differentiation capacities coexist in the bone marrow. HSCs were, therefore, classified into three subsets depending on their differentiation capacities to produce either lymphoid cells (lymphoid-biased), myeloid cells (myeloid-biased), or both (balanced). As HSCs age, the myeloid-biased HSCs accumulate at the expense of the lymphoid-biased HSC subset (Muller-Sieburg et al. 2012). This compositional shift towards the enlarged myeloid-biased HSCs may explain the age-associated increase in HSC population with myeloid skewing and the lymphoid lineage deficiency in the elderly (Cho et al. 2008). Moreover, the CD150 marker expression can be used to distinguish between the subsets of murine HSCs (Beerman et al. 2010): myeloid-biased cells and balanced HSCs express CD150 at high or lower level, respectively. Furthermore, differentiation and self-renewal programs are epigenetically stable in each HSC subset. This means that pre-programmed differentiation fate is stably inherited within the HSC clone. For example, a myeloid-biased HSC will give rise to myeloid-biased daughter stem cells throughout the life of the organism. Notably, the lifespan of a clone determines the representation of the particular HSC subset. Therefore, in this case, the myeloid-biased HSCs may have a higher self-renewal capacity than the lymphoid counterpart, leading to myeloid dominance at an advanced age (Muller-Sieburg et al. 2004). It should be noted that HSCs have a broad differentiation capacity and these cells can give rise not only to myeloid and lymphoid cell lineages but also to several other tissue cell types (Ogawa et al. 2013). However, to the best of our knowledge, there is currently no information on how the age related alterations affect the pluripotent nature of HSCs.

#### HSCs and their microenvironment

Along with the age-associated hematopoietic cell-intrinsic changes, including lineage potential and HSCs lifespan, it should be emphasized that HSCs have a special home in the bone marrow (the HSC niche) and the interactions between the HSCs and their microenvironment are also altered with age (Van Zant and Liang 2012).

HSCs are located in intimate contact with their surrounding niche/stromal cells, localized mainly around the sinusoidal/endothelial cells and in the endosteal region of the bone marrow (Shen and Nilsson 2012). Importantly, HSCs receive several surviving stimuli from their niche cells. This is supported by the long standing observation that HSCs rapidly loose stem cell properties ex vivo, i.e., without their physiological microenvironment. What are the extrinsic niche-derived signals that influence the aging process in the hematopoietic system? The full picture is not clear yet. The accumulating evidence indicate that the age-related changes in HSC microenvironment and the niche signals, along with the intrinsically regulated differentiation processes, have a strong impact on HSC properties (Wagner et al. 2008).

Age-associated alterations in the bone marrow composition include the general loss of bone mass due to decreased bone formation, accumulation of fat cells, altered extracellular matrix elements and reduced transforming growth factor (TGF)-beta levels (Bellantuono et al. 2009). It is not clear yet and remains to be established how the aged niche influences the stem cell population. In elderly people, HSCs have a reduced ability to home the niche upon bone marrow transplantation and in elderly people the HSC can be mobilized more easily after granulocyte-colony stimulating factor (G-CSF) administration than in young individuals (Geiger et al. 2007). HSC’s homing and mobilization processes depend on cell adhesion molecules. Therefore, it is not surprising that integrins (α4, α5) and the cell adhesion molecule, VCAM-1, are expressed at higher levels in young than in old HSCs, supporting the notions that the aged HSCs adhere less efficiently to the aged stromal cells and that the cross-talk between HSCs and the microenvironment is compromised (Xing et al. 2006). Interestingly, in vivo the old progenitor cells are located more distant from the endosteal niche cells in the bone marrow than young cells, suggesting that the altered niche-HSC synapses play a role in the observed age-related changes in the stem cell homing and the mobilization (Kohler et al. 2009).

### HSCs as pathophysiological determinants in aging

Age-related changes in HSCs may be responsible for multiple pathological conditions in the elderly population (Rothstein 2003; Signer et al. 2007). The decline of the lymphoid cell production leads to impaired immune defense and higher susceptibility to infections. The consequence of the smaller lymphocyte population is the reduced number of B and T cell clones, leading to the production of less diverse antibody and T-cell receptor repertoires with age. Under these conditions, the probability to acquire autoimmune diseases greatly increases. Along with these effects, the enlarged but functionally impaired myeloid cell population creates a pro-inflammatory milieu in the body. As the immune cells are not localized in one defined area in the body, the age-related pro-inflammatory environment and the compromised immunosurveillance also favor the emergence of non-hematopoietic cancers (von Figura and Rudolph 2009). The most detrimental aspect of aging in the hematopoietic system is however the exponential increase of leukemias with age (Dombret et al. 2008; Vas et al. 2012). Moreover, whereas childhood malignancies predominantly involve lymphoid leukemia, the leukemias in the elderly have mainly myeloid origin, reflecting the age-associated myeloid skewing of the HSC population (Henry et al. 2011; Rossi et al. 2008).

In addition to the age-associated hematopoietic disorders mentioned above, anemic status with low erythrocyte count in the blood is very common in elderly. One of the difficulties in studying age-related anemia is that in many cases, the de facto cause of inefficient red blood cell production is unclear and, therefore, they are referred as “unexplained anemia in the elderly” (UAE) (Merchant and Roy 2012). One possible explanation is that the levels of erythropoietin, a survival factor of the erythroid precursors, is relatively low in UAE patients and consequently the red blood cell production is reduced.

### Future perspectives

The two main consequences of HSC aging—the decline in their functional capacities and imbalance in blood cell production—may greatly contribute to organismal aging and the development of age-related pathology. Some of the age-associated effects, such as the accumulation of genomic insults and telomere shortening, are generally considered irreversible, whereas other effects, such as epigenetic modifications and signaling pathway switches, are potentially reversible (Mendelsohn and Larrick 2013; Pollina and Brunet 2011).

With this in mind, valuable efforts have been undertaken to rejuvenate old HSCs. For example, a recently discovered Cdc42 inhibitor molecule (CASIN) was able to functionally rejuvenate old murine HSCs. Cdc42 is a small RhoGTP-ase that by regulating the cytoskeletal organization is involved in several signalling pathways and epigenetic processes, including genome markings by histones (Lugert et al. 2010). Following CASIN treatment, the lymphoid cell production of old HSCs increased and the epigenetic status (measured by H4K16Ac level) of the old HSCs was restored (Florian et al. 2012). Along the same line of evidence, restoring the activity of a mammalian sirtuin SIRT3, shown to be reduced in aged murine HSCs, rejuvenated these cells as demonstrated by in vitro colony forming assay and in vivo transplantation experiments (Brown et al. 2013). SIRT3 reduced oxidative stress, known to alter histone deacetylase (HDAC) activity (Cyr and Domann 2011). Taken together, these data support the concept that age-related epigenetic pathways can be reversed leading to a rejuvenated phenotype in HSCs.

The regenerative capacity of HSC transplantation (HSCT) provides an alternative approach for the treatment of age-related hematological malignancies. In HSCT, cells are collected from mobilized blood, bone marrow or umbilical cord blood and intravenously injected in the preconditioned recipient (Gupta et al. 2011). The well-established limitation of HSCTs is the insufficient number of stem cell donors. Moreover, the potential donor list is further narrowed only to the young population, because HSCs isolated from older donors engraft much less efficiently in the recipient bone marrow than younger HSCs that results in poorer marrow function (Anasetti 2008). We, therefore, predict that methods that enhance HSC engraftment in the new bone marrow microenvironment and procedures that contribute to the HSC expansion will improve the efficiency of HSC transplantation in the elderly.

## Neural stem cells

In 1962, Altman showed for the first time that the adult mammalian brain can generate new neurons (Altman 1962). Since then, many researchers have addressed this topic and today we can unequivocally state that neurogenesis in mammals continues throughout life. The importance and impact of the newly generated cells on the adult CNS function have not been clearly established, however, rodent experiments indicate that the adult-born neurons may play a role in the regulation of olfaction, mood, learning and memory (Gould et al. 1999; Garthe et al. 2009; Lehmann et al. 2013; Lazarini and Lledo 2011; Goodman et al. 2010; Sahay et al. 2011). However, despite the efforts and interest in the field, the picture of adult neural stem cells is still obscure. As mentioned above, stem cells in general are defined by their ability to self-renew and give rise to differentiated progenies. The neural stem cells (NSCs) in the adult brain have not yet been proven to be *bona fide* stem cells, since there is an apparent heterogeneity in the cell populations at multiple stages of adult neurogenesis (Lugert et al. 2010; Cho et al. 2008; Conboy and Rando 2005; Decarolis et al. 2013).

The two main regions in the adult brain that give rise to new neurons are the subventricular zone (SVZ) lining the lateral ventricles and the subgranular layer (SGL) of the hippocampal dentate gyrus (Fuentealba et al. 2012). The cells formed in the SVZ assemble into a chain called rostral migratory stream (RMS) that, upon reaching the olfactory bulb (OB), gets anatomically and functionally incorporated into the OB network (Belluzzi et al. 2003). The neuronal precursors of the SGL, however, differentiate locally and form connections within the hippocampal circuitry (van Praag et al. 2002).

The localization, morphology and behavior of the NSCs greatly differ between the SVZ and SGL. Nevertheless, similarities between these two populations during the process of the generation of new neurons can be observed [reviewed in (Ming and Song 2011)]. In both regions, the NSCs possess radial glia-like properties, being GFAP- and Nestin-immunoreactive. These cells are in a quiescent state and upon activation they proceed to a transit-amplifying/progenitor phase in which they rapidly proliferate. Subsequently, these cells give rise to doublecortin (DCX)-expressing neuroblasts that eventually turn into mature NeuN-positive neurons. The new neurons are generated in a special environment called the stem cell niche, which contains ependymal cells, astrocytes, microglia (resident macrophages of the CNS) and blood vessels (Fuentealba et al. 2012).

### Age-related changes in NSCs

In this section we summarized recent studies on the regulation of adult NSC maintenance and differentiation, with particular focus on aging.

#### NSC pool and proliferation potential

It is now generally accepted that the production rate of adult-born neurons decreases with age in the mouse SVZ and SGL (Maslov et al. 2004; Lugert et al. 2010). Despite the decline in the number of total NSCs, the percentage of actively mitotic NSCs increases with age, suggesting that other downstream processes (such as altered cell survival or differentiation potential) should account for the reduction of new-born neurons in old mice (Shook et al. 2012; Stoll et al. 2011b).

#### Age-related changes in human neurogenesis

The extent and functional relevance of human neurogenesis is still under debate. It has been shown that in humans there is limited postnatal neurogenesis in the olfactory bulb (Bergmann et al. 2012) and neurogenesis in the neocortex is restricted to perinatal age only (Bhardwaj et al. 2006). Also, neurogenesis in the SVZ and the formation on migrating chain of neuroblasts (RMS) can be observed only in infants and are basically missing in the adult (Sanai et al. 2011).

Earlier studies from Eriksson et al. (1998) showed that in post-mortem human hippocampus, BrdU (thymidine analog labeling the DNA in S phase administered to the individuals before their death) co-localizes with neuron-specific markers, suggesting the generation of new neurons even in middle-aged and aged persons. Later studies confirmed these findings and completed the picture with the observations that qualitative and quantitative age-related changes in the hippocampus are similar in humans and rodents (Knoth et al. 2010). In vivo MRI studies revealed age-dependent shrinkage of the human hippocampus [reviewed in (Ho et al. 2013)]. Whether the change in the morphology of this brain region corresponds to decreasing neuron production is not clear yet.

Marmosets (New World primate) display adult neurogenesis both in the SGL and SVZ, and aging is associated with the decrease of DCX-positive neuroblasts (Bunk et al. 2011; Leuner et al. 2007). However, the number of neuroblasts was lower in the marmoset than in age-matched mouse, further supporting the idea that evolutionary more developed species such as humans may not possess extensive adult neurogenesis (Bunk et al. 2011).

#### Systemic regulators

Very elegant heterochronic parabiosis experiments, in which old and young mice (parabionts) were connected in different combinations via their circulation, demonstrated that factors present in the blood can affect neurogenesis, synaptic plasticity and spatial learning (Villeda et al. 2011). Young parabionts showed reduced neurogenesis in the hippocampus, while impaired neurogenesis in old mice was partially rescued. The chemokine CCL11 was identified as one of the mediators of this process in old mice. Other chemokines, such as MCP-1 and MIP1, also regulate the maintenance of adult NSCs cultured in vitro and contribute to different effects on young and middle-aged animals (Gordon et al. 2012).

#### The NSC niche

Several signaling pathways and factors are known to modulate neurogenesis in the adult and aged CNS [reviewed in (Artegiani and Calegari 2012; Riddle and Lichtenwalner 2007)]. Here, we will list only some of them supported by recent evidence to give a hint on the several factors at work.

Interestingly, age-related decrease in hippocampal neurogenesis is not associated with a decline in the expression of selected genes important for NSC proliferation and neurogenesis in the dentate gyrus (DG) (Shetty et al. 2013). However, endothelial cells in the stem cell niche express more TGF-beta1 in aged mice than in young adults, which provokes enhanced quiescence and apoptosis of the NSCs (Pineda et al. 2013). Furthermore, astrocytes located in the niche secrete VEGF and FGF2 to promote the NSC maintenance. During aging, the activity of these cells decline, which results in decreased growth factor production and subsequent drop in the level of neurogenesis (Bernal and Peterson 2011). Astrocytes are also involved in the regulation of adult NPC proliferation by mediating Wnt signaling.

In aging, decreased astrocytic activity and cross-talk lead to reduced Wnt signaling and maintenance of NSCs (Miranda et al. 2012). Restoring the down-regulated levels of Survivin (an Inhibitor of Apoptosis protein and a downstream target of Wnt), results in enhanced NPC proliferation in the aging brain. Sox2 also regulates the expression of Survivin and the apoptosis and the survival of NSCs in the SVZ (Feng et al. 2013). Reduction of Wnt levels affects the expression of other target genes, as well, e.g. NeuroD and DCX that are regulated in a reversible way in an exercise-dependent manner (Okamoto et al. 2011). In the hippocampus, the levels of Wnt antagonist Dkk1 increase with age, accounting for the reduced Wnt signaling. In agreement with these findings, old Dkk1 mutant mice show improved memory consolidation and affective behaviour (Seib et al. 2013). Besides astrocytes, microglial cells are also thought to modulate the Wnt-mediated NSC proliferation via the secretion of proinflammatory factors in the neurogenic niche (L’Episcopo et al. 2013). Metabolic changes (such as decreased mitochondrial content and oxygen consumption) can also account for altered neural stem cell behavior and new neuron production in the nervous system of aged animals (Stoll et al. 2011a).

In sum, the altered microenvironment and signaling in the NSC niche lead to a reduced rate of neurogenesis in old age.

### NSCs in age-related neurodegeneration

Given the presence and potential functional significance of adult-born neurons in the CNS, diseases affecting the nervous system may impinge on or be modulated by the newly emerging cells from the neurogenic regions. The most common age-related neurodegenerative disorders manifested in the brain are Alzheimer’s disease (AD), Huntington’s disease (HD) and Parkinson’s disease (PD). The main features of these pathologies involve aberrant protein aggregation, selective neuronal loss, and subsequent physical and mental decline. There are several genetic rodent models established to investigate the pathogenesis of the devastating diseases [e.g. (Elder et al. 2010) for AD; (Cepeda et al. 2010) for HD, and (Magen and Chesselet 2010) for PD]. Although in most cases these models do not completely reflect the phenotypes observed in patients, they serve very valuable subjects to decipher some basic aspects of the cellular changes occurring during AD, HD or PD. In the next section, we summarize the recent progress in the regulation of adult NSCs in neurodegenerative disease models.

#### Alzheimer’s disease

Alzheimer’s disease can be characterized by (i) the altered processing of the transmembrane amyloid precursor protein (APP) resulting in the accumulation of extracellular amyloid-beta deposits (senile plaques), and (ii) the hyperphosphorylation of the microtubule-associated protein tau that aggregates in the cell to neurofibrillary tangles. The most affected brain regions/cell types in AD are the basal forebrain cholinergic neurons, hippocampus and cortex.

In AD model systems, NSCs of different transgenic animals respond differently depending on the type of transgene [reviewed in (Lazarov and Marr 2010)]. In some models, the NSC proliferation and differentiation potential increase, whereas in other models, the opposite effect is observed. In humans, the picture is also far from being clear: some research groups showed elevated levels of DCX and neuron-specific markers (Jin et al. 2004), while others reported a decrease in DCX-positive cell number via bone morphogenetic protein 6 (BMP6) signaling (Crews et al. 2010) in the hippocampus of AD patients compared to non-demented controls. All these discrepancies observed both in animal and human studies may be due to diversity in the utilized transgenic models, tests performed at different ages or different stages of the disease. Therefore, further detailed and systematically comparable studies are required to draw clear conclusions of the relationship between AD and neurogenesis. Intriguingly, longitudinal studies in humans and experiments performed in AD transgenic mice have revealed that enhanced social, mental and physical activity (enriched environment) in elderly people/aged animals protects against dementia and AD (Fratiglioni et al. 2004; Arendash et al. 2004; Jeong et al. 2011). It would be attractive to speculate that these protective effects are mediated by NSCs.

#### Huntington’s disease

Mutations in the huntingtin gene observed in HD cause the precipitation of the encoded protein product that leads to the damage and elimination of medium spiny GABAergic neurons in the striatum and other neurons in the cortex.

The HD mouse models suggested that the accumulation of the mutant protein impairs neurogenesis in the hippocampus (Fedele et al. 2011). Remarkably, enriched environment (Lazic et al. 2006; Spires et al. 2004; Sullivan et al. 2001), but not physical exercise (Kohl et al. 2007), could compensate for the reduced number of neurons. Histological analyses of HD patients showed increased neurogenesis in the striatum (Curtis et al. 2003) but not in the hippocampus [reviewed in (Ransome et al. 2012)], suggesting that adult-born neuronal progenitors can arise close to the striatum and migrate to the damaged area to replace the lost cells. Similarly, when GABAergic neurons were ablated in the murine striatum, SVZ progenitors migrated to the lesioned region and gave rise to NeuN-positive mature neurons (Tattersfield et al. 2004). These data indicate that upon neuronal cell death, NSC-derived cells can be attracted to fill the gap in the neural tissue.

#### Parkinson’s disease

In PD, the misfolded alpha-synuclein in the dopaminergic neurons forms intracellular aggregates that provoke the selective cell death of the midbrain dopaminergic neurons. The most prominent clinical feature of PD is the dysregulation of motor functions (muscle rigidity, tremor), however, other non-motor phenotypes appear, as well (e.g. cognitive dysfunction, depression).

Animal and human studies of Parkinson’s disease gave more or less clear-cut evidence for impaired SVZ and SGL neurogenesis [reviewed in (Mochizuki 2011; Marxreiter et al. 2013)]. Although the exact mechanisms underlying the reduced NSC proliferative and differentiation potential in PD are still unknown, recent data suggest that disturbed Wnt/beta-catenin signaling pathway mediated by microglial cells could be at work (L’Episcopo et al. 2013).

Interestingly, in patients suffering from all above listed diseases, olfactory deficit is exhibited even before the main symptoms (Jimbo et al. 2011; Lazic et al. 2007; Doty 2012). This raises the possibility of the involvement of adult NSCs derived from the SVZ or the SGL in the pathogenesis of the disorders.

### Future perspectives

The age-related alterations in NSCs are under extensive debate for several reasons. First, neither marker nor morphology analyses could identify a single NSC population in the neurogenic regions of the adult brain. In addition, the extent of adult neurogenesis seems to be quite limited, therefore, its relevance in the adult brain functions is fairly controversial. Finally, it should be noted that most studies have been carried out in rodent models while the anatomy and physiology of the rodent and human neuronal networks show some differences. Therefore, the interpretation of the experimental results needs careful consideration.

Nevertheless, a great body of data has accumulated over the years about the mechanisms, regulation, and role of adult neurogenesis and its changes during aging. This knowledge may be exploited to overcome some ARDs affecting the nervous system (such as Alzheimer’s, Parkinson’s or Huntington’s disease). One way to replace the lost cells in the brain is to transplant neurally committed cells to the damaged area. This procedure, however, raises serious concerns about the fate of the injected cells, the extent of regeneration and its relevance in the above mentioned diseases, since the pathological phenotype of the diseased endogenous cells may affect the graft tissue, as well. Another therapeutic avenue, which seems less invasive, is to activate the endogenous stem cells located in the nervous system to replace the eliminated neurons and glial cells. Nevertheless, the limited availability of adult NSCs may interfere with this approach to cure devastating diseases. Further studies in the field will hopefully shed more light on the process of adult neurogenesis and will enable the development of promising and effective strategies to cure at least some age-related neurodegenerative diseases.

## Mesenchymal stem cells

Mesenchymal stem cells (MSCs) were identified almost forty years ago by the work of Friedenstein et al. (1976). They described a rare population of cells that developed into colony forming units following plastic adherence of bone marrow cells. MSCs are bone marrow derived, non-hematopoietic cells that differentiate into cells of the mesenchymal lineage, including bone, cartilage, adipose, tendon and muscle tissue. Additionally, MSCs can give rise to ectoderm-type cells, e.g. neuron-like cells (Dezawa et al. 2004), and endoderm-like cells, e.g. hepatocytes (Lee et al. 2004). Notably, one of the challenges in the identification of MSCs is the absence of specific surface markers. The MSCs can be identified by various markers (such as CD13, CD29, CD31, CD44, CD54, CD63, CD73, CD 90, CD105, CD106, CD140b, CD166, Stro1), along with the absence of the hematopoietic markers (Colter et al. 2000; Pittenger et al. 1999). The properties of MSCs such as their ease of culture expansion, immunomodulatory activity, and differentiation potential make these cells potentially ideal candidates for tissue engineering and cell replacement therapies.

### Age-related changes in MSCs

Since MSCs may provide a major source of cells for therapeutic purposes, age-related alterations with respect to their pool size and function have been extensively addressed.

#### MSC pool and proliferation potential

Reports about the effect of aging on isolated human MSC proliferation are conflicting. In a number of studies, no significant changes in cultured MSC doubling time or proliferation capacity have been observed in young versus elderly people (Scharstuhl et al. 2007; Suva et al. 2004; Stenderup et al. 2001). In contrast, several research groups suggested that increasing age has a negative impact on MSC growth (Alt et al. 2012; Mareschi et al. 2006; Zaim et al. 2012), cell number and performance (Stolzing et al. 2008). The observed discrepancies might be due to the fact that different laboratories apply various methods to derive and culture MSCs (Sethe et al. 2006). As long as the protocols are not standardized for MSC isolation, identification, and maintenance, it will be difficult to draw clear-cut conclusions about the impact of aging on MSCs’ properties.

#### MSC differentiation potential

Results surrounding the age-related differentiation capacity of MSCs, mostly osteogenic differentiation, are also conflicting (Sethe et al. 2006). Several reports showed a decrease in osteogenic potential of MSCs with age. For example, Moerman et al. (2004) suggested that aging increases the commitment of MSCs to the adipocyte lineage at the expense of the osteoblast lineage. However, others reported no change (Roura et al. 2006; Oreffo et al. 1998). Interestingly, a study by Murphy et al. (2002) showed a reduction in chondrogenesis in MSCs from osteoarthritis patients, suggesting that the changes in the differentiation capacity of the cells are the underlying cause for the increase in bone mass and the loss of cartilage in osteoarthritis.

### The use of MSCs in the therapy of age-related diseases

MSCs may provide an ideal source for transplantation, considering that their isolation and application is well established, and they can be isolated from various adult tissues.

#### Diabetes

Diabetes has become one of the major causes of illness and death in the elderly (Sima et al. 2009). A large number of studies have reported the application of MSCs in both type I and type II diabetes. Under certain conditions, bone marrow derived MSCs from diabetes patients can differentiate into functional islet pancreatic cells, suggesting that these MSCs may be potentially used as a source of autologous insulin-producing cells for beta-cell replacement (Sun et al. 2007). Also, there is supporting evidence for the use of allogeneic or xenogeneic MSCs in pancreatic regeneration as demonstrated by the positive effects of islet/bone marrow transplantation in a monkey model (Berman et al. 2010). In addition to regeneration mechanisms, the paracrine effects of MSCs could play an important role in the treatment of diabetes as demonstrated by in vivo and in vitro studies (Hu et al. 2009; Xu et al. 2011). The transplantation of MSCs could enhance regeneration of endogenous progenitor cells, probably via the secretion of a variety of factors, such as insulin growth factor-1 (IGF-1), vascular endothelial growth factor (VEGF), and basic fibroblast growth factor (bFGF) (Xu et al. 2011).

#### Cardiovascular diseases

The age-related changes in cardiomyocyte structure and function are a major risk factor for cardiovascular disease (Sheydina et al. 2011). The acute myocardial infarction (AMI), which is caused by the obstruction of the blood supply to the heart with subsequent loss of cardiomyocytes, is the leading cardiac disease, mostly occurring in the old population. Early studies offered therapeutic promise for the application of MSC engraftment in AMI (Pittenger et al. 1999; Makino et al. 1999), presumably due to the release of cytokines and growth factors that stimulate endogenous repair mechanisms (Amado et al. 2005; Kucia et al. 2004; Tang et al. 2004). Recent progress from experimental animal studies and clinical trials supported the beneficial effects of MSCs in AMI treatment. It was further suggested that the MSCs act by releasing paracrine signals, by differentiating into cardiomyocytes, smooth muscle cells, and vascular endothelial cells, and by stimulating endogenous repair (Boyle et al. 2010).

#### Neurodegenerative diseases

MSCs can pass the blood–brain barrier, therefore, they can be delivered to the nervous system without invasive surgical methods (Momin et al. 2010). The use of MSCs in the treatment of neurodegenerative diseases, particularly in fatal and difficult to treat disorders, such as AD and PD (see previous section), is under debate. Nonetheless, current data supports that MSC transplantation is safe and may be used to slow down the progression of neurodegenerative diseases (Uccelli et al. 2011; Mezey 2007; Olson et al. 2011; Spees et al. 2006). For example, previous reports demonstrated that MSCs survive following transplantation into different mouse models, can differentiate into neural cells and promote recovery of brain function (Mezey 2007). They act at least in part by secreting factors that support injured neurons and promote the survival and the regeneration of host cells. Taken together, the existing data support MSC-based cellular therapies for neurodegenerative disorders (Mitrecic 2011). However, extensive research and clinical trials are required to gain further insights into the underlying mechanisms responsible for the positive effects of MSC transplantation and to determine the efficacy of such treatments.

### Future perspectives

MSCs possess several key characteristics that make them potentially ideal cells for cellular therapies toward aging and ARDs. Depending on their tissue of origin, MSCs are biased in the type of cells they give rise to (Porada and Almeida-Porada 2010). Thus special care needs to be taken in respect to the specific disease and the target organ. It is reasonable to assume that variable clinical effects may result from the diverse methods employed for isolating and culturing MSCs, differing levels of contaminating cells and the duration of the culture. Moreover, ARDs are complex, challenging and not entirely understood, supporting that their treatment will involve multiple, interdisciplinary approaches. Much work remains to be done, including basic science to further understand the connection between MSC aging and the major ARDs as well as clinical trials to explore their potential clinical use.

## Skeletal muscle stem cells

The skeletal muscle satellite cells, or myogenic stem cells, were first identified and described in 1961 by two independent studies by Mauro (1961) and Katz (1961). These cells reside between the myofiber plasmalemma and the basal lamina. Large body of research conducted since Mauro’s and Katz’s seminal discoveries showed that these cells are responsible for myofiber growth and repair [reviewed in (Yablonka-Reuveni 2011)]. The satellite cells were also suggested to play a role in myofiber hypertrophy during adult life, although this issue still remains controversial (McCarthy and Esser 2007; O’Connor and Pavlath 2007).

Skeletal muscle satellite cells are quiescent under normal physiological conditions. In response to various cues, such as exercise, injuries or disease, they expand, proliferate as myoblasts or undergo myogenic differentiation to fuse and restore damaged muscle. Notably, skeletal muscle satellite cells are sustained throughout adult life due to repeated cycles of growth and regeneration, underlying their self-renewal properties and qualifying them as tissue stem cells (Brack et al. 2007; Collins et al. 2005; Collins and Partridge 2005; Sacco et al. 2008). Recently, a distinct subpopulation of satellite cells has been shown to represent a reversible dormant stem cell state and generate distinct daughter cells by asymmetric DNA segregation during muscle regeneration (Rocheteau et al. 2012). Over the years, additional stem cell populations with myogenic capacity have been identified [reviewed in (Pannerec et al. 2012)]. Here, we will mainly focus on satellite stem cells, which are the major skeletal muscle progenitor cells having indispensable role in myofiber regeneration.

### Age-related changes in skeletal muscle satellite cells

What happens to skeletal muscle satellite cells with advanced age? As in most tissues, the regenerative capacity of satellite cells is reduced. It seems that satellite cells are different from other tissue stem cells since they do not lose their ability to participate in tissue maintenance and repair, but are rather devoid of the optimal environmental cues required for regeneration (Conboy et al. 2005; Conboy and Rando 2005).

#### Pool of satellite cells

The abundance of resident satellite cells is still under debate. Whereas some suggested that satellite pool is relatively constant during aging and may vary, depending on the specific muscle examined or the method used (Brack and Rando 2012), others demonstrated a decline that occurs soon after birth and then further along with age (Shefer et al. 2013). This reduction is more striking in females and can be attenuated by exercise (Day et al. 2010; Shefer et al. 2013; Phelps et al. 2013). Notably, it was recently suggested that satellite cells comprise two distinct populations: one, which is responsible for muscle maintenance throughout life and the number of which decreases with age, and second, which is activated by severe muscle injury, survives transplantation and remains at constant quantities from birth to old age (Neal et al. 2012).

#### Regenerative capacity of satellite cells

If indeed the number of clones that produces reserve cells is reduced with age, then it is possible that satellite cell depletion is responsible for the reduced capacity to generate a reserve population (Day et al. 2010). Moreover, additional factors beyond satellite cell activity play a role in reduced muscle repair in old age (Shavlakadze et al. 2010). This notion is supported by the observation that the myogenic stem cell response is transiently delayed following engraftment as a result of delayed inflammation, however, the final regeneration response is proper (Shavlakadze et al. 2010). Indeed, factors in the systemic environment profoundly regulate the stem cell niche as shown by the rejuvenation of skeletal stem cells activity using cross-transplantation (Carlson and Faulkner 1989; Conboy et al. 2005) or parabiosis (Conboy et al. 2005) of young and old mice. Conversely, a regenerative phenotype typical of old age was evident when young mice were injected with serum obtained from old mice (Brack et al. 2007). This age-related systemic effect is associated with an alteration in the myogenic differentiation potential, expressed as increased fibrogenic lineage potential leading to increased fibrosis (see below).

#### Systemic regulators of satellite cells

Given the importance of systemic factors in the control of muscle regeneration, their signaling pathways have been extensively studied. A pioneering study by Conboy et al. (2003), demonstrated that Notch signaling is a key factor of muscle regenerative potential that declines with age. Notch interplays with the age-associated excessive production of TGF-beta, which induces unusually high levels of pSmad3 and hampers satellite cell regeneration (Carlson et al. 2008). Moreover, Notch is required for the maintenance of the quiescent state of muscle stem cells by regulating self-renewal and differentiation (Bjornson et al. 2012). Satellite cell pool is maintained by Notch since the deletion of recombining binding protein-Jκ (RBP-Jκ) (required for Notch signaling) results in depletion of stem cells and muscles lacking any ability to regenerate in response to injury (Bjornson et al. 2012).

Factors in the systemic environment of old animals mediate the conversion of satellite cells from myogenic to fibrogenic lineage (Brack et al. 2007). These processes are associated with increased Wnt signaling, leading to the activation of the downstream effectors Axin2 and β-catenin. Conversely, inhibition of Wnt restores muscle regeneration in old mice. It was later reported that GSK3-beta orchestrates a temporal switch between Notch to Wnt signaling that regulates proliferative expansion and subsequently differentiation during postnatal myogenesis (Brack et al. 2008). The involvement of Wnt in the regulation of regenerative potential of muscle cells was further demonstrated by the work conducted on the Wnt receptor Fzd7 and its candidate ligand Wnt7a (Le Grand et al. 2009). Wnt7a promotes the symmetric expansion of satellite cells without affecting the growth or differentiation of myoblasts and controls satellite cell number following regeneration. The systemic role of Wnt in satellite muscle regeneration has been challenged by showing that Wnt activity was not present in sera, did not contribute to the TGF-beta1-dependent inhibition of satellite cell myogenic potential and did not improve the regeneration of old muscle in vivo (Carlson et al. 2009).

### Satellite cells in muscle disease therapy and future perspectives

Sarcopenia, the loss of muscle fibers at an advanced age, is one of the major causes for falls and subsequently health deterioration in the elderly. The underlying reason for the reduced muscle mass is not entirely clear, and the contribution of age-related changes in satellite stem cell is under debate (Pallafacchina et al. 2012; Shadrach and Wagers 2011). Most other muscle diseases including the muscular dystrophies are genetic disorders associated with progressive muscle weakness and degeneration. In many of these diseases, myofibers are readily damaged and their satellite cell mediated repair leads to the exhaustion of the satellite cell pool. There is currently no cure for muscular dystrophies; however, satellite cell (or myotube) transplantation may provide a promising strategy to slow disease progression and improve the patient’s quality of life by enhancing muscle repair [recently reviewed in (Briggs and Morgan 2013)]. Given that genetic muscle disorders are not affected by aging classifies them out of the scope of this review. Nonetheless, understanding the effects of aging on satellite cells may provide important insights into transplantation strategies and lead to the identification of therapeutic targets.

Satellite stem cells seem to be “more resistant” to aging than other tissue stem cells (Conboy et al. 2005) and cells from older animals maintain their regeneration properties in a young environment. We therefore speculate that transplantation of satellite cells will provide superior results compared to other types of stem cells with respect to the proportions of functional transplanted cells. Moreover, the satellite cells are markedly affected by their niche, supporting that transplantation of these cells in young patients will result in better outcomes than in old patients. It would be interesting to directly check this hypothesis. Clearly, transplantation success does not only depend on the functionality of the cells; several challenges, such as the donor to host histocompatibility or cell migration, need to be overcome. Moreover, there are additional complications that are involved in myofiber regeneration specifically, such as the requirement to deliver the cells to many host muscles at the same time. Last but not least, limited understanding of the niche of satellite cells requires further investigation to assure that the cells will be provided with the required environment, including pro-inflammation and stromal cells (Briggs and Morgan 2013).

Several issues concerning the age-related effects on satellite cells remain a mystery. The following questions should be addressed: (1) Will satellite cells derived from young animals or humans result in higher therapeutic success? (2) Will the age of the host influence the functionality and thus the success rates of the transplantation? Further investigation of the regulators that are at work during aging will likely allow increasing the success rates of cell-based therapies and thus improving the life quality of muscular disease patients.

## Concluding remarks

The association between adult stem cells dysfunction and aging has been subjected to extensive research. Here, we emphasize that aging manifests differently in adult stem cells depending on their tissue of origin (Table 1). For example, differentiation potential of HSCs is hampered by differentiation bias as demonstrated by the accumulation of myeloid-biased cells (Muller-Sieburg et al. 2012). In the case of satellite cells, the decreased differentiation potential resulted from a conversion into a fibrogenic lineage (Brack et al. 2007). Another important point is the interplay between the tissue stem cells and their microenvironment. In NSCs, the age-related decrease in hippocampal neurogenesis is associated with altered signals received from the niche endothelial cells and astrocytes (Pineda et al. 2013; Bernal and Peterson 2011). On the other hand, the dysfunction of blood forming stem cells with age is thought to be mainly attributed to intrinsic regulation (Xing et al. 2006). It is likely that HSCs are regulated by cues arising from the aged stroma cells as well, resulting in enhanced mobilization and reduced homing of aged HSCs.

Advancing current knowledge in age-related alterations in adult stem cell contributes to our understanding of not only the pathophysiological alterations occurring in ARDs, but also of the use of stem cells in regeneration medicine. As discussed herein, aging tissue stem cells are involved in the initiation or progression of diseases in the elderly, such as in the cases of HSCs in leukemias, NSCs in neurodegenerative diseases and MSCs in osteoarthritis. Further studies characterizing the involvement of stem cells and their complex interactions with the microenvironment in the disease are vital. Such studies are expected to provide new insights into how to modulate the age-related processes in stem cells and may allow inhibiting or delaying the disease progression. Last but not least, special care needs to be taken when considering adult stem cells for regenerative therapies since stem cell function highly depends on the microenvironment, and the aging or imbalanced niche negatively affects stem cell performance (Larsen et al. 2012). Thus, a deeper understanding of the in vivo stem cell regulation will likely contribute to the development of novel in vitro techniques to model, isolate and propagate stem cells in a reliable manner to maintain their capacities for potential use in human therapy.
